# The Association of Microplastics in Peripheral Blood and Pulmonary Disease: A Pilot Study

**DOI:** 10.3390/jox16030072

**Published:** 2026-04-26

**Authors:** Scott A. Helgeson, Hossny Alaws, Mohamed I. Ibrahim, Augustine S. Lee, Danielle H. W. Vlecken, Hassan Z. Baig

**Affiliations:** 1 Division of Pulmonary Medicine, Department of Medicine, Mayo Clinic, 4500 San Pablo Road S, Jacksonville, FL 32224, USA; alaws.hossny@mayo.edu (H.A.); baig.hassan@mayo.edu (H.Z.B.); 2 SV Biotech BV, Robert Boyleweg 4, 2333 CG Leiden, The Netherlands; daniellevlecken@gmail.com

**Keywords:** microplastics, biomonitoring, blood, chronic lung disease, case-control study

## Abstract

Microplastics may pose health risks, particularly for chronic lung diseases. Clarifying the link between circulating microplastics and pulmonary disease is vital for shaping research and interventions. The objective of this study was to evaluate whether microplastics in peripheral blood are associated with COPD or IPF vs. no lung disease. In this pilot prospective case-control study, participants were grouped as control (*n* = 10), COPD (*n* = 9), or IPF (n = 10). Relevant comorbidities and exposures were obtained from records and questionnaires. All underwent standardized blood collection (PlasticTox©). Samples were analyzed for total microplastic concentration, stratified by size (<10 µm, 10–30 µm, 30–70 µm). The primary outcome was to show a difference in total microplastic burden between lung disease and controls. Secondary measures were to determine size-specific concentrations and associations with demographic variables and smoking history. Among 29 participants (median age 70 (IQR 64–80); 14 women (48.3%)), COPD/IPF groups had significantly higher total microplastic concentrations vs. controls (median 26.0 vs. 3.5 particles/100 µL; *p* < 0.01). Particle burdens <10 µm and 10–30 µm were particularly elevated (both *p* < 0.01). After adjusting for smoking, only the <10 µm fraction remained independently associated with lung disease (adjusted odds ratio 1.94 (95% CI, 1.23–7.04)). In this pilot exploratory study, individuals with COPD or IPF showed greater circulating microplastic levels than controls. These findings should be interpreted as hypothesis-generating, and larger analytically validated studies are needed to clarify directionality, causal mechanisms, contamination control, and the clinical relevance of circulating microplastic burden.

## 1. Introduction

Widespread use of plastic began in the 1950s and has continued to increase, with production rising from 0.5 million tons per year in 1960 to 348 million tons per year by 2017 [1,2,3,4]. Recent studies have suggested that atmospheric fallout in urban areas is a measurable source of microplastics inhalation, with an estimated 3–10 tons of microplastics deposited annually [5].

The widespread presence of microplastics in the environment has garnered increasing attention because of their potential adverse effects on human health. Microplastics (MPs) are plastic particles less than 5 mm in size, and nanoplastics (NPs) have a size of 0.1 µm [6]. Microplastics and nanoplastics have been identified in various environmental matrices, including air, water, and soil [7,8]. Human exposure can occur through ingestion, inhalation, and direct skin contact [9]. Inhalation is considered a major route of exposure relevant to pulmonary health [7,10]. Human biomonitoring studies have reported detectable particles in blood, lung tissue, sputum, bronchoalveolar lavage fluid, and other enclosed body fluids, although reported concentrations vary widely across studies because of major differences in sampling, contamination control, and analytical methods.

Studies have identified microplastics in various organ systems including heart, lungs, gastrointestinal tract, and reproductive organs, which have been associated with organ dysfunction [3,11,12,13]. For example, elevated microplastic levels in carotid plaques have been associated with higher adverse cardiovascular events [12]. Inhalation of airborne microplastics particularly in occupational settings has been associated with chronic pulmonary inflammation and fibrosis as well as other airway disorders like asthma. Pneumoconioses have been linked with certain exposures to polyvinyl chloride particles and polymer resins from engineered stone, for example, and identification of these particles within the lung parenchyma and airways. Some of the mechanisms implicated in the effect of microplastics on lung disease include disruption of the respiratory tract epithelium, direct cytotoxicity on pulmonary epithelial cells, increased release of pro-inflammatory mediators, oxidative stress, and redox imbalance, as well as increasing the effect of environmental allergens by disrupting the alveolar epithelial barrier [4].

Despite the growing body of in vitro evidence, there is a notable lack of in vivo research. The biokinetics of MPs in the human body, including their uptake, distribution, retention, and excretion, remain poorly understood [14]. Human biomonitoring studies have detected microplastics in the lower airway using techniques like bronchoalveolar lavage fluid and blood-based samples, but direct comparisons between circulating microplastic burden and chronic lung disease phenotypes remain limited [14].

The unique value of this study is that it compares circulating microplastic concentrations in patients with two chronic pulmonary diseases, IPF and COPD, with concentrations in a control group without lung disease using the same collection platform and size-stratified reporting approach. We hypothesized that patients with COPD or IPF would have higher circulating microplastic concentrations, particularly in smaller size fractions, than controls without lung disease. Because the available human literature remains methodologically heterogeneous, this pilot study was designed as an exploratory, hypothesis-generating investigation to inform larger analytically validated studies.

## 2. Materials and Methods

### 2.1. Study Design

This was a prospective observational case-control pilot study designed to examine the association between circulating microplastics and lung disease in patients with IPF or COPD compared with subjects without lung disease. This study was approved by the IRB. The study was performed from 17 May 2024 to 21 November 2024. The STROBE reporting format utilized was from the EQUATOR network. Because this was an observational case-control study without an assigned intervention, as defined by the NIH, it was not registered as a clinical trial. Generative AI was not used for this study.

### 2.2. Study Population

Patients were identified from an academic pulmonary clinic and were divided into three groups: (1) no pulmonary disease labeled as “control” for this study; (2) a diagnosis of idiopathic pulmonary fibrosis (IPF); or (3) diagnosis of chronic obstructive pulmonary disease (COPD). The control group had no pulmonary disease, defined by the absence of parenchymal lung disease as evidenced by a CT scan of their chest and a normal complete pulmonary function test within 2 months of the blood sampling. IPF diagnosis was made by a multidisciplinary interstitial lung disease group [15]. COPD was diagnosed by a pulmonologist based on respiratory symptoms, clinical context, patient history, and airflow obstruction on pulmonary function tests [16,17].

Participants were enrolled with a pragmatic target of approximately 10 patients per group to provide pilot feasibility data and preliminary effect-size estimates for future studies; no formal a priori sample size calculation or case-control matching strategy was used. All patients that were screened and approached to participate in the study agreed and had their blood samples taken. After the patients consented for the study, a chart review of electronic medical records was performed to identify comorbidities. In addition, we screened the patients with the CHEST Interstitial and Diffuse Lung Disease Patient Questionnaire, which was used to obtain information on pulmonary and exposure history [18].

### 2.3. Sample Collection

The same procedures and sampling techniques were followed for all groups to ensure consistency. Informed consent was obtained from all participants before any part of this study was performed.

We utilized the PlasticTox© (Grand Blanc, MI, USA) kit, a commercially available direct-to-consumer microplastics detection kit, to collect peripheral blood samples and analyze the presence of microplastics particles. This kit is not FDA approved, and the present study did not perform independent analytical validation against reference methods such as µFTIR, Raman spectroscopy, or pyrolysis-GC/MS, as this study was not evaluating the test itself. The kit included bandages, blood lancets, sterile gauze, an alcohol prep pad, a five-spot blood paper card, a PlasticTox© insert, a demographics card, and a sample shipping envelope. A sample of dried human blood was obtained by a finger pick using metal lancets and placed on the five-spot blood card. The performing laboratory was blinded to clinical groups and provided no identifiable information other than the blood sample. The sample was then mailed to the laboratory and tested to look for microplastics particles such as polyester (PES), polyethylene terephthalate (PET), polyamides (PA), polypropylene (PP), polyethylene (PE), polyurethane (PU), polyvinyl chloride (PVC), and polystyrene (PS) [19,20,21,22]. For analysis, the samples were eluted, vortexed and incubated at 37 °C. Subsequently, the samples were reconstituted in and incubated with a Nile Red solution (CAS:7385-67-3) as Nile Red has been previously described as a substance that stains many plastics, including PE (Supplemental Figures S1 and S2) [19,20,21,22]. The results were qualitatively confirmed with microscopy. The samples containing PE were applied to glass fiber filters and were stained with Nile Red and observed under a microscope, both brightfield and with fluorescent light [19,20,21,22]. These particles were stratified by their size. The sample was tested for the total number of these microplastic particles per 100 microliters of blood and by size distribution (<10 micrometers, 10–30 micrometers, and 30–70 micrometers). The blood sample did not contact any plastics in collection, delivery, or analysis. However, formal procedural blanks, field blanks, and airborne background contamination monitoring were not performed in this pilot study, which is an important limitation. The lab performing the test is clinical laboratory improvement amendment (CLIA) certified.

### 2.4. Data Analysis

Continuous data were displayed as median with interquartile range, and categorical data were displayed as number with percentage. Comorbidity or survey data were described descriptively. For univariate analysis of particle data (total number of particles, and particle size breakdown), a Spearman correlation test was used for continuous variables, and for 2-group comparisons of particle counts, either the Wilcoxon rank-sum test or for multiple group comparisons, the Kruskal–Wallis test was used. Pairwise group comparisons were performed using two-sided Mann–Whitney U tests for total microplastic concentration and each size fraction. Given the small sample size, the multivariable logistic regression model was intentionally parsimonious and exploratory; adjusted odds ratios for lung disease were computed using smoking history and particle size fractions as covariates. A stacked bar chart was generated by summing particle counts within each size fraction for each diagnostic group and expressing these as proportions of the total microplastic count within that group, providing a descriptive visualization of size distribution rather than an inferential statistical comparison. No minimum detection-frequency threshold was prespecified in this pilot study. A *p* value <0.05 was considered statistically significant. Data were analyzed using Bluesky© v10.3.1 (Chicago, Il, USA).

## 3. Results

A total of 29 participants were included in the final analysis (10 control, 9 with COPD, and 10 with IPF). One COPD participant’s blood sample was lost in transport and was excluded. The median age was 70.5 years (IQR, 64.5–76.5) in the control group, 70.0 years (IQR, 60–80) in those with COPD, and 77.0 years (IQR, 69–85) in those with IPF (Table 1). Female participants were more prevalent in the COPD group (*n* = 6, 66.7%) than in the control (*n* = 4, 40%) and IPF (*n* = 4, 40%) groups. All participants in the control and IPF groups identified as white, whereas the COPD group included two (22.2%) black participants. Body mass index (BMI) values were similar across groups. Smoking history was most common in the COPD group, with eight (88.9%) former smokers, compared with three (30%) in the control group, and five (50%) in the IPF group. Comorbidities, including diabetes mellitus, coronary artery disease, peripheral vascular disease, chronic kidney disease, congestive heart failure, autoimmune disorders, cancer, and neurologic disease, were represented in all groups but did not appear to cluster disproportionately in any single category.

Family history of lung disease was more common among participants with COPD (*n* = 7, 78%) compared with those in the control (*n* = 0) or IPF (*n* = 3, 30%) groups. Relevant exposure history is summarized in Table 2, and full survey results are in Appendix A. Living in an old house within the prior 10 years and the presence of potential mold sources were reported across all groups (control, *n* = 3 (30%); IPF, *n* = 3 (30%); and COPD, *n* = 1 (11%) and 2 (22%), respectively). Occupational and environmental exposures varied, but COPD participants reported having lived or worked in environments with heavy smoke or dust (*n* = 4, 44%) more frequently compared with either control (1 (10%)) or IPF (4 (40%)) participants. IPF participants had higher rates of reported silica (2 (20%)) and asbestos (2 (20%)) exposure relative to other groups. These exposure variables were collected descriptively and were not included in multivariable modeling because of the small sample size.

All but one patient, who was in the control group, had microplastics detected in their blood with a positive rate of detection of 96.5%. The range of total microplastic burden ranged from 0 µm per 100 µL to 68 µm per 100 µL. In univariate analyses (Table 3), the presence of lung disease was associated with higher total microplastic concentration (*p* < 0.01), as well as with the <10-µm (*p* < 0.01) and 10 to 30 µm (*p* < 0.01) size categories, but not with the 30 to 70 µm category (*p* = 0.21). Only three participants had measurable particles in the 30 to 70µm category; accordingly, results for this size bin should be interpreted with particular caution and are best considered descriptive. Other demographic factors, including age, gender, race, body mass index, and pack-year smoking history, were not significantly correlated with any microplastic size category. The only exception was a negative correlation between years since quitting smoking and microplastics in the 10 to 30 µm range (Spearman ρ = −0.55; *p* = 0.03).

**Figure 1:**
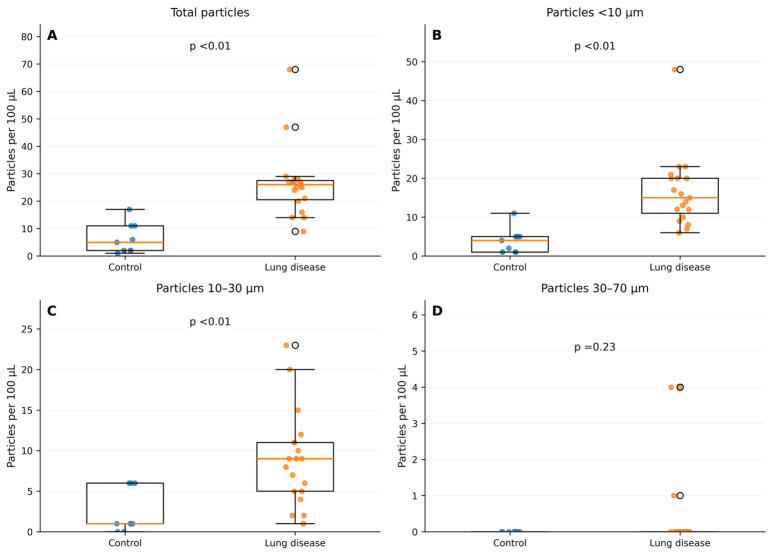
Control versus combined lung disease. A box whisker plot comparing the microplastic concentration between the control and combined lung disease groups. (**A**) total microplastic concentrations; (**B**) microplastic concentration <10 µm; (**C**) microplastic concentration 10–30 µm; and (**D**) microplastic concentration 30–70 µm.

When comparing control participants with participants with lung disease, there were associations with total particle concentration (*p* < 0.01), particle size <10 µm concentration (*p* < 0.01), and particle size 10–30 µm concentration (*p* < 0.01) (Figure 1). The larger size category (30–70 µm) was not associated with lung disease (*p* = 0.23).

**Figure 2:**
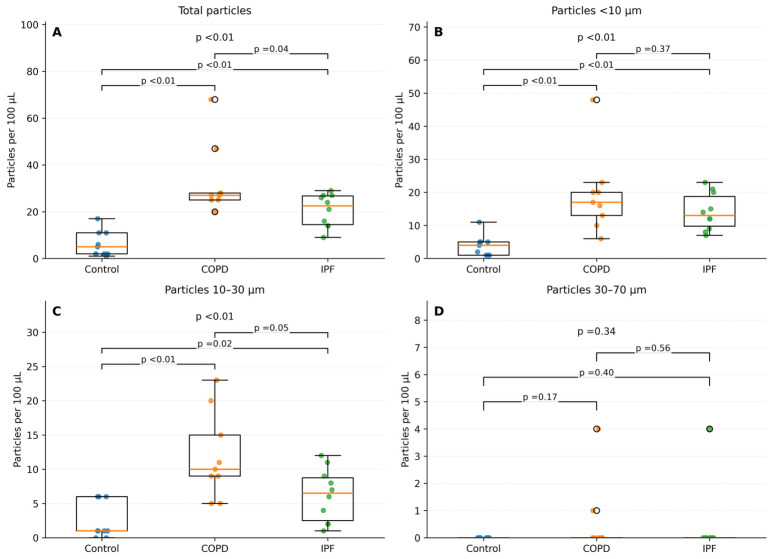
Comparison across diagnostic groups (control *n* = 9). A box whisker plot comparing the microplastic concentration between the control, chronic obstructive pulmonary disease and idiopathic pulmonary fibrosis groups. (**A**) total microplastic concentrations; (**B**) microplastic concentration <10 µm; (**C**) microplastic concentration 10–30 µm; and (**D**) microplastic concentration 30–70 µm.

When lung disease was separated into COPD and IPF, both groups showed higher total microplastic concentration (COPD, *p* < 0.01; IPF, *p* < 0.01), microplastic concentration < 10 µm (COPD, *p* < 0.01; IPF, *p* < 0.01), and microplastic concentration 10–30 µm (COPD, *p* < 0.01; IPF, *p* < 0.01) compared with controls (Figure 2). There was no association with 30–70 µm particles (COPD, *p* = 0.17; IPF, *p* = 0.40).

**Figure 3:**
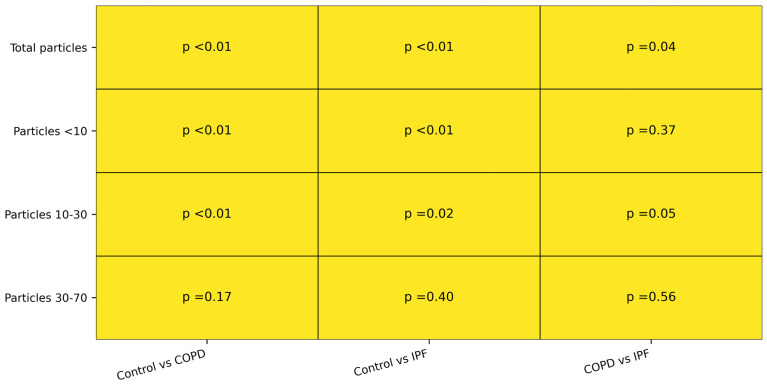
Pairwise *p* value summary. This figure summarizes pairwise comparisons of circulating microplastic concentrations between controls, patients with COPD, and patients with IPF for total particles and each particle-size fraction. Pairwise comparisons were performed using Mann–Whitney U tests, and *p* values are displayed for each group comparison. The strongest between-group differences were observed for total microplastic concentration and the smaller particle fractions, particularly in comparisons involving controls, whereas differences between COPD and IPF were less pronounced.

**Figure 4:**
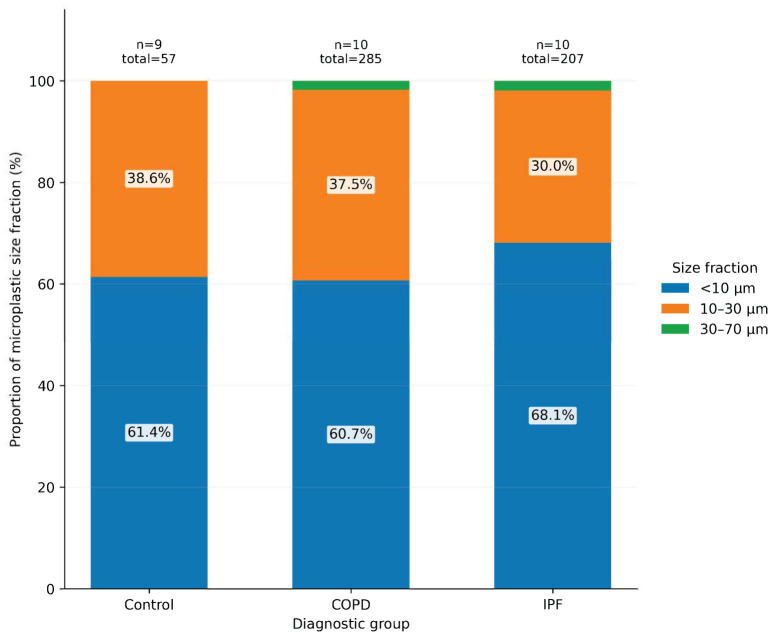
Proportional distribution of microplastic size fractions. This stacked bar chart illustrates the relative contribution of each particle-size fraction (<10 µm, 10–30 µm, and 30–70 µm) to the total circulating microplastic burden within each diagnostic group. Smaller particles accounted for the majority of detected microplastics across all groups, with the <10 µm fraction representing the largest proportion in controls, COPD, and IPF. The 30–done70 µm fraction contributed minimally within each group, consistent with its low overall detection frequency.

Pairwise testing demonstrated that the differences in circulating microplastic burden were between controls and the disease groups, especially for total particles and the smaller size fractions (Figure 3). In contrast, comparisons between COPD and IPF showed less separation, suggesting that elevated circulating microplastics may be a shared feature across these chronic lung diseases rather than disease-specific to one diagnosis. The proportional size distribution was broadly similar across groups, with most particles falling in the <10 µm and 10–30 µm ranges and only a very small contribution from particles measuring 30–70 µm (Figure 4).

In an exploratory logistic regression model adjusted for smoking history and particle size fractions, only the microplastic concentration in the <10 µm size range remained associated with the presence of lung disease (odds ratio 1.94; 95% CI, 1.23–7.04). Given the small sample size and risk of model overfitting, this result should be interpreted as hypothesis-generating rather than confirmatory.

## 4. Discussion

This pilot study found that patients with COPD or IPF had higher circulating microplastic concentrations than controls without lung disease, with the largest differences observed in the <10 µm and 10–30 µm size fractions. Microplastic particles were also detected in controls, but at lower levels. To our knowledge, this is among the first investigations to compare blood-based microplastic burden in established chronic lung diseases with a control population using a uniform collection system. The findings should be interpreted as exploratory and hypothesis-generating rather than mechanistic or causal, but they carry important implications for understanding both disease mechanisms and future clinical management strategies.

Microplastic pollution and its interaction with human health has garnered heightened attention in recent years [23,24,25]. While initial investigations focused on the environmental presence of microplastics, emerging data now indicates that human biological tissues, including lung tissue, placental tissue, and blood, can harbor plastics [5,26,27,28,29]. A prior study reported the presence of microplastics in human blood, supporting the premise that systemic exposure can be measured in living humans and may be more pervasive than previously anticipated [28]. Direct quantitative comparison between prior studies and the present study is difficult, because methods, units, contamination controls, and particle-identification platforms differ substantially across reports. That heterogeneity is itself important and underscores the need for standardized human biomonitoring approaches.

Findings of elevated numbers of small microplastic particles (<10 µm and 10–30 µm) in patients with lung disease aligns with mounting evidence that it is biologically plausible because smaller particles may be more likely to cross pulmonary barriers and enter the bloodstream [30,31,32,33]. Microplastics in the <10 µm range are particularly concerning given their propensity for cellular uptake and translocation into systemic circulation [34,35]. Previous in vitro work suggests that particles smaller than 20 µm can be internalized by alveolar epithelial cells, raising the possibility of an enhanced inflammatory response and tissue remodeling in susceptible individuals [36]. COPD and IPF are both characterized by chronic inflammation and structural changes in the lung that may exacerbate the translocation of inhaled particles into the systemic circulation. At the same time, the reverse explanation is equally plausible: pre-existing lung disease may impair clearance or barrier function and thereby permit greater particle accumulation in the blood. The present data cannot distinguish between these possibilities.

Several potential mechanisms could explain why patients with COPD and IPF demonstrated higher circulating microplastic levels. Compromised mucociliary clearance in COPD may allow inhaled microparticles to persist in the respiratory tract and eventually translocate across the alveolar epithelium [37]. Fibrotic changes in IPF may disrupt normal alveolar–capillary barrier function, facilitating particle passage into the bloodstream [27,28,29]. Chronic inflammation could further increase vascular and epithelial permeability, providing additional routes for microplastic entry [30,31]. Although these mechanisms are plausible, they remain speculative in the context of this cross-sectional pilot study and should be tested in longitudinal and mechanistic investigations.

The observed signal may also reflect broader exposure patterns rather than disease-specific biology alone. Chronic exposure to and accumulation of microplastics has been associated with inflammatory responses, oxidative stress, and cytotoxic effects in experimental models [38]. While the exact health impacts of microplastics remain debated, there is growing concern that these particles may exacerbate pre-existing pulmonary and systemic conditions [7]. From a clinical standpoint, patients with COPD and IPF are already vulnerable to complications such as acute exacerbations, cardiovascular events, and reduced quality of life [15]. This study did not measure dietary intake, ambient pollution, indoor dust, socioeconomic status, or other environmental determinants of exposure, and it did not assess urinary, biliary, or other excretory pathways. Accordingly, our findings should not be interpreted to mean that circulating microplastics contribute directly to acute exacerbations, lung function decline, or disease progression. Those questions require larger longitudinal studies with clinical outcome tracking.

All three groups in this study showed similar smoking histories based on median pack-year. Yet, the lung disease groups demonstrated elevated microplastic concentrations relative to controls, suggesting that while tobacco use may be one conduit for plastic exposure, it is unlikely to fully explain the increased microplastic burden observed in individuals with lung disease. Cigarette filters are frequently composed of cellulose acetate, a plastic that can fragment into microplastics, which may represent one route of exposure [39,40]. Several studies have identified microplastics in the lungs of long-term smokers, raising the question of increasing inflammatory processes in chronic lung disease [13,27,41,42]. Although the groups had differing smoking distributions, smoking history alone did not appear to explain the observed pattern, and the <10 µm fraction remained associated with lung disease in the exploratory adjusted model. An additional finding was the inverse correlation between years since smoking cessation and circulating microplastic concentration in the 10–30 µm fraction. Although this association should be interpreted cautiously given the small sample size and exploratory nature of the analysis, it may suggest that more remote smoking exposure is associated with lower intermediate-sized circulating particle burden over time or show an ability for excretion of microplastics after exposure. Residual confounding is highly likely because smoking is only one of many relevant exposure variables, and the sample size did not allow robust multivariable adjustment for occupational, environmental, dietary, inflammatory, or comorbidity-related factors.

From a clinical perspective, these data should primarily increase awareness that circulating microplastics can be detected in patients with chronic lung disease and may warrant further study as an exposure or biomonitoring signal. At present, the findings do not justify changes in disease management. Instead, they support future work incorporating validated analytical techniques, rigorous contamination control, polymer-specific characterization, and longitudinal assessment of clinical outcomes in COPD and IPF.

Several limitations merit discussion. First, the small single-center sample size precludes definitive conclusions about causality or temporal relationships. It limits precision, generalizability, and the ability to perform robust multivariable analyses or meaningful subgroup comparisons between COPD and IPF. Second, the cross-sectional design precludes causal inference and cannot distinguish exposure-driven disease from disease-driven particle accumulation. It remains unclear whether elevated microplastic levels contribute to lung disease pathogenesis or simply accumulate in diseased patients secondary to impaired clearance or increased accumulation through the lungs. Third, the PlasticTox©/Nile Red-based approach is not FDA approved, was not independently validated in this study, and does not provide spectroscopic confirmation of polymer identity; therefore, these results should be interpreted as semi-quantitative proxy measures rather than definitive measurements of microplastic burden. Fourth, procedural blanks, field blanks, and airborne background contamination monitoring were not performed, and fingertip blood sampling itself may be vulnerable to contamination despite efforts to avoid plastic contact. Fifth, polymer-specific concentrations, detection frequencies, and formal excretion or retention measurements were not available. Sixth, multiple comparisons were not corrected, the 30–70 µm size range had very low detection frequency, smoking and exposure histories were self-reported, and residual confounding by environmental, dietary, occupational, socioeconomic, inflammatory, and comorbidity-related factors is likely. We also did not collect data regarding vaping history for this study. These limitations reinforce that the present findings are preliminary and should serve as justification for larger, analytically rigorous studies.

## 5. Conclusions

In conclusion, this pilot study identified higher circulating microplastic counts, particularly in smaller size fractions, in patients with COPD or IPF compared with controls without lung disease. These data support an association between chronic lung disease and blood-based microplastic burden but do not establish directionality or causality. Future studies should use analytically validated detection methods, rigorous contamination control, larger and more diverse cohorts, polymer-specific characterization, and longitudinal clinical follow-up to determine the biological and clinical significance of these findings.

## Figures and Tables

**Table 1:** Patient characteristics and comorbidities.

	Control *n*= 10	Chronic Obstructive Pulmonary Disease *n* = 9	Idiopathic Pulmonary Fibrosis *n* = 10
Gender			
Female	4 (40%)	6 (66.7%)	4 (40%)
Male	6 (60%)	3 (33.3%)	6 (60%)
Age	70.5 (64.5 to 76.5)	70.0 (60 to 80)	77.0 (69 to 85)
Race			
White	10 (100%)	7 (77.8%)	10 (100%)
African American	0	1 (11.1%)	0
Caribbean Black	0	1 (11.1%)	0
BMI	25.5 (24 to 27)	23.9 (20.8 to 27.1)	22.4 (18.9 to 25.9)
Smoking status			
Current	0	0	0
Former	3 (30%)	8 (88.9%)	5 (50%)
Never	7 (70%)	1 (11.1%)	5 (50%)
Pack-years	21	25 (13.5 to 36.5)	23 (1.5 to 44.5)
Comorbidities			
DM	2 (20%)	3 (33.3%)	1 (10%)
CAD	2 (20%)	3 (33.3%)	3 (30%)
PVD	1 (10%)	3 (33.3%)	1 (10%)
CKD	1 (10%)	3 (33.3%)	1 (10%)
CHF	1 (10%)	2 (22.2%)	2 (20%)
Autoimmune	1 (10%)	0	0
Cancer	3 (30%)	3 (33.3%)	0
Neurologic disease	4 (40%)	5 (55.6%)	4 (40%)

Data are displayed as count and percentage or median with IQR. DM = diabetes mellitus, CAD = coronary artery disease, PVD = peripheral vascular disease, CKD = chronic kidney disease, CHF = congestive heart failure.

**Table 2:** Pulmonary and exposure history.

	Control *n* = 10	Chronic Obstructive Pulmonary Disease *n* = 9	Idiopathic Pulmonary Fibrosis *n* = 10
Have you lived in an old house within the past 10 years?	3 (30%)	1 (11%)	3 (30%)
Does your current or past home or workplace have any of the following?			
Humidifier	3 (30%)	3 (33%)	1 (10%)
Sauna	1 (10%)	1 (11%)	0 (0%)
Hot tub/Jacuzzi	2 (20%)	1 (11%)	3 (30%)
Water damage	1 (10%)	1 (11%)	1 (10%)
Mold	1 (10%)	2 (22%)	3 (30%)
Animals	5 (50%)	5 (55%)	7 (70%)
Birds	0 (0%)	1 (11%)	1 (10%)
Have you lived or worked in an environment where you were exposed to heavy smoke or dust?	1 (10%)	4 (44%)	4 (40%)

Data are displayed as count with percentage.

**Table 3:** Univariate analysis of demographic data of entire cohort with microplastic concentration by total counts and size distribution.

	Total Microplastics per 100 µL	Plastic Size <10 µm per 100 µL	Plastic Size 10–30 µm per 100 µL	Plastic Size 30–70 µm per 100 µL
Age	r = −0.31 (*p* = 0.24)	r = −0.02 (*p* = 0.93)	r = −0.30 (*p* = 0.25)	r = −0.31 (*p* = 0.24)
Gender (M vs. F)	16.0 (5.5 to 27.5) vs. 22.5 (13.75 to 31.25); *p* = 0.71	11.0 (3.5 to 18.5) vs. 13.0 (6.5 to 19.5); *p* = 0.66	6.0 (2.0 to 10.0) vs. 6 (1.5 to 10.5); *p* = 0.48	0 vs. 0; *p* = 0.56
Race (W vs. B)	17.0 (6.5 to 27.5) vs. 26.5; *p* = 0.24	11.0 (3.5 to 18.5) vs. 14.5; *p* = 0.55	6.0 (2.0 to 10.0) vs. 12.0; *p* = 0.13	0 vs. 0; *p* = 0.68
BMI	r = 0.21 (*p* = 0.43)	r = 0.45 (*p* = 0.08)	r = −0.20 (*p* = 0.46)	r = 0.09 (*p* = 0.73)
Pack-year smoking history	r = −0.041 (*p* = 0.88)	r = −0.16 (*p* = 0.56)	r = 0.16 (*p* = 0.56)	r = 0.01 (*p* = 0.98)
Years since quit smoking	r = −0.45 (*p* = 0.08)	r = −0.35 (*p* = 0.18)	r = −0.55 (*p* = 0.03)	r = −0.33 (*p* = 0.22)
Lung disease presence (yes vs. no)	26.0 (18.0 to 34.0) vs. 3.5 (0 to 8.0); *p* < 0.01	16.0 (12.0 to 20.0) vs. 3.0 (1.0 to 5.0); *p* < 0.01	9.0 (6.0 to 12.0) vs. 1.0 (0 to 4.0); *p* < 0.01	0 vs. 0; *p* = 0.21

Table shows Spearman correlation coefficients with *p* values. Data are presented as median particles per 100 µL with IQR or as otherwise described in the row heading. A median and IQR could not be calculated for the 30–70 µm category because only a small number of participants had particles in this size range. An IQR could not be calculated for race because only 2 participants were in the Black group. M = male, F = female, W = White, B = Black.

## Data Availability

The data presented in this study are available on request from the corresponding author due to the possibility of containing information that could compromise the privacy of research participants.

## Supplementary Materials

The following supporting information can be downloaded at: https://www.mdpi.com/article/10.3390/jox16030072/s1, Figure S1: Control sample; Figure S2: Sample showing microplastics.

## Abbreviations

The following abbreviations are used in this manuscript:

COPD	Chronic obstructive pulmonary disease
IPF	Idiopathic pulmonary fibrosis

## Appendix A

**Table A1:** Full survey results.

	Control N = 10	Chronic Obstructive Pulmonary Disease N = 9	Idiopathic Pulmonary Fibrosis N = 10
Do any of your grandparents, parents, brothers, sisters, aunts, uncles, cousins, or children have any of the following lung diseases?			
Emphysema, COPD	0 (0%)	7 (78%)	3 (30%)
Asthma	0 (0%)	2 (22%)	1 (10%)
Sarcoidosis	0 (0%)	0 (0%)	0 (0%)
Cystic fibrosis	0 (0%)	0 (0%)	0 (0%)
Pulmonary fibrosis	0 (0%)	0 (0%)	1 (10%)
Hypersensitivity pneumonitis	0 (0%)	0 (0%)	0 (0%)
Have you lived in an old house within the past 10 years?	3 (30%)	1 (11%)	3 (30%)
Does your current or past home or workplace have any of the following?			
Humidifier	3 (30%)	3 (33%)	1 (10%)
Sauna	1 (10%)	1 (11%)	0 (0%)
Hot tub/Jacuzzi	2 (20%)	1 (11%)	3 (30%)
Water damage	1 (10%)	1 (11%)	1 (10%)
Mold	1 (10%	2 (22%)	3 (30%)
Animals	5 (50%)	5 (55%)	7 (70%)
Birds	0 (0%)	1 (11%)	1 (10%)
Have you previously lived outside this country?	1 (10%)	3 (33%)	2 (20%)
Have you lived or worked in an environment where you were exposed to heavy smoke or dust?	1 (10%)	4 (44%)	4 (40%)
Have you ever performed any of the following occupations?			
Farm work	1 (10%)	1 (11%)	0 (0%)
Painter	0 (0%)	1 (11%)	0 (0%)
Sand blaster	0 (0%)	0 (0%)	0 (0%)
Pipe fitter	0 (0%)	1 (11%)	0 (0%)
Automotive mechanic	0 (0%)	0 (0%)	0 (0%)
Welder	0 (0%)	0 (0%)	0 (0%)
Insulator	0 (0%)	1 (11%)	0 (0%)
Vineyard worker	0 (0%)	0 (0%)	0 (0%)
Carpenter	1 (10%)	2 (22%)	1 (10%)
Laboratory worker	0 (0%)	0 (0%)	0 (0%)
Longshoreman	0 (0%)	0 (0%)	0 (0%)
Have you ever worked in any of the following locations?			
Mine	0 (0%)	0 (0%)	0 (0%)
Quarry	0 (0%)	0 (0%)	0 (0%)
Pulp mill	0 (0%)	0 (0%)	0 (0%)
Bakery	0 (0%)	0 (0%)	0 (0%)
Foundry	0 (0%)	0 (0%)	0 (0%)
Railroad	0 (0%)	0 (0%)	0 (0%)
Paper mill	0 (0%)	0 (0%)	0 (0%)
Smelting	0 (0%)	0 (0%)	0 (0%)
Plastic factory	0 (0%)	0 (0%)	0 (0%)
Tunnel construction	0 (0%)	0 (0%)	0 (0%)
Have you ever been exposed to the following at work/ home/ elsewhere?			
Birds	1 (10%)	0 (0%)	0 (0%)
Feathers	1 (10%)	0 (0%)	0 (0%)
Fishmeal	0 (0%)	0 (0%)	0 (0%)
Insecticide	1 (10%)	0 (0%)	0 (0%)
Fertilizer	1 (10%)	0 (0%)	0 (0%)
Beryllium	0 (0%)	0 (0%)	0 (0%)
Cobalt	0 (0%)	0 (0%)	0 (0%)
Tin	0 (0%)	0 (0%)	0 (0%)
Iron oxide	0 (0%)	0 (0%)	0 (0%)
Aluminum	0 (0%)	1 (11%)	0 (0%)
Mica	0 (0%)	0 (0%)	0 (0%)
Silica	0 (0%)	0 (0%)	2 (20%)
Asbestos	0 (0%)	1 (11%)	2 (20%)
Coal	0 (0%)	0 (0%)	0 (0%)
Cheese	0 (0%)	0 (0%)	1 (10%)
Maple bark	0 (0%)	0 (0%)	0 (0%)
Wheat	0 (0%)	0 (0%)	0 (0%)
Coffee/ tea	0 (0%)	0 (0%)	1 (10%)
Mushroom	0 (0%)	0 (0%)	0 (0%)
Oil	0 (0%)	0 (0%)	0 (0%)
Sugar cane	0 (0%)	0 (0%)	0 (0%)
Malt	0 (0%)	0 (0%)	0 (0%)
Meat	0 (0%)	0 (0%)	0 (0%)
Cotton	0 (0%)	0 (0%)	0 (0%)
Wood	0 (0%)	0 (0%)	1 (10%)
Industrial strength cleaning solution	0 (0%)	0 (0%)	0 (0%)
Oily nose drops	0 (0%)	0 (0%)	0 (0%)
Cork	0 (0%)	0 (0%)	0 (0%)
Detergent	0 (0%)	0 (0%)	0 (0%)
Pottery	0 (0%)	0 (0%)	0 (0%)
Talc	0 (0%)	0 (0%)	0 (0%)
Paint	0 (0%)	1 (11%)	0 (0%)
Cement	0 (0%)	1 (11%)	2 (20%)
Pipes	0 (0%)	0 (0%)	1 (10%)
Brakes	0 (0%)	0 (0%)	0 (0%)
Tile	0 (0%)	0 (0%)	1 (10%)
