# Supplementary material for: The Association of Microplastics in Peripheral Blood and Pulmonary Disease: A Pilot Study
Source: J Xenobiot. 2026 Apr 26;16(3):72. doi: 10.3390/jox16030072 (PMC13214817; doi:10.3390/jox16030072)

Journal of Xenobiotics

Supplementary Materials: The association of microplastics in peripheral blood and pulmonary disease: a pilot study

Scott A Helgeson; Hossny Alaws; Mohamed I. Ibrahim;Augustine S. Lee; Danielle H.W. Vlecken; Hassan Z. Baig

Figure S1: Control sample showing no microplastics

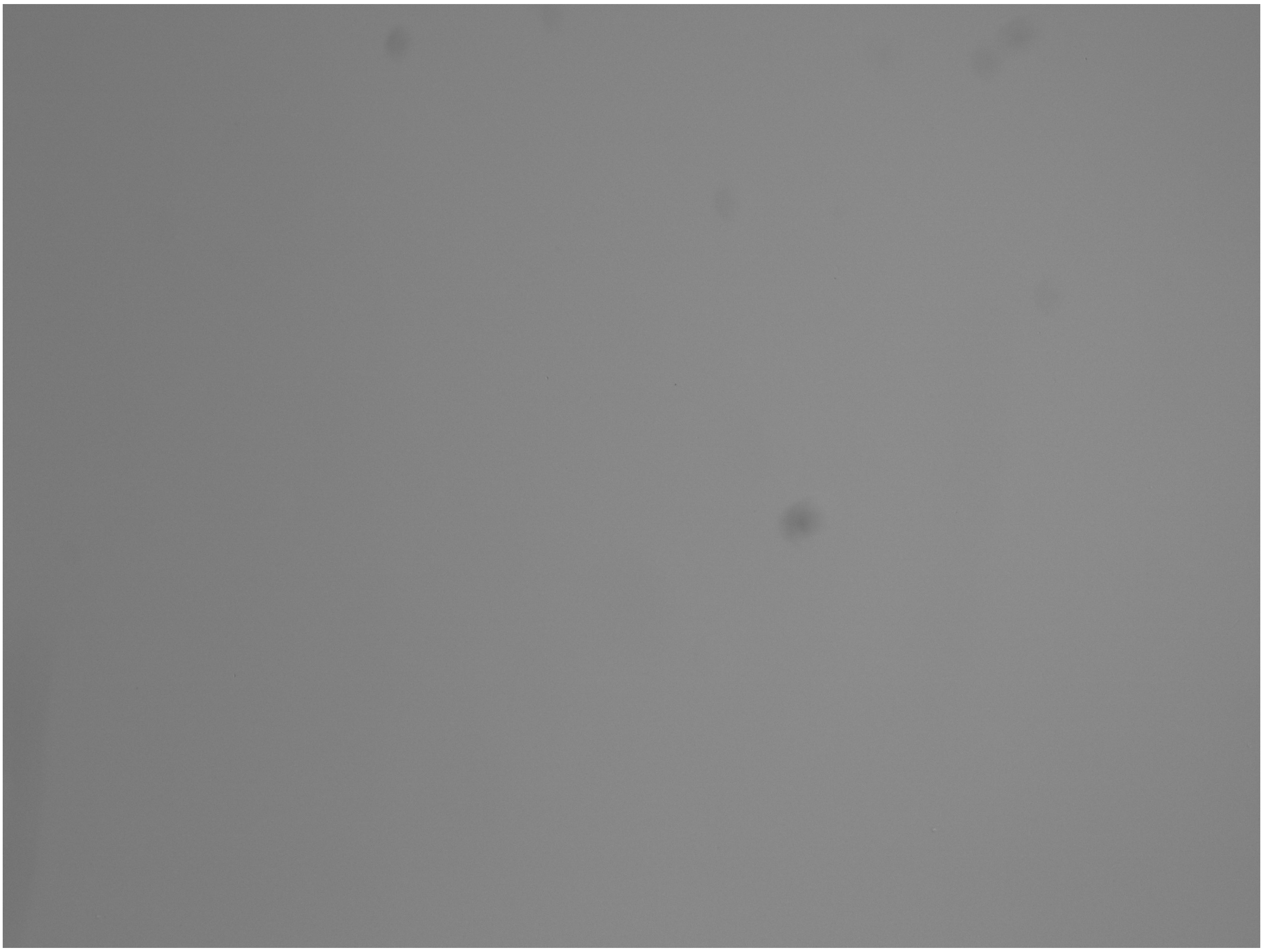

Figure S2: Patient's sample showing microplastics

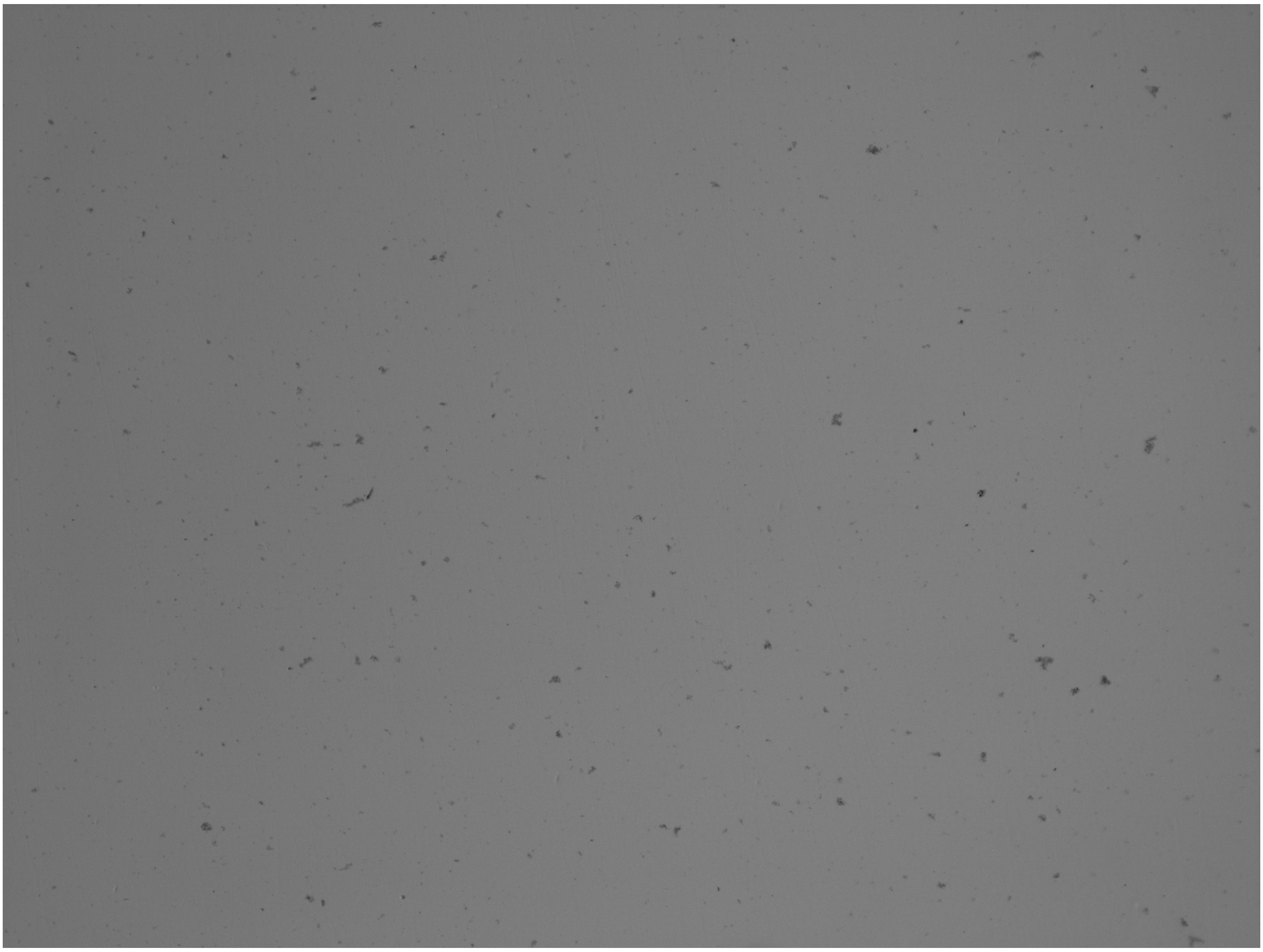

Supplement: Supplementary file 1 [file jox-16-00072-s001.zip › jox-4226238-supplementary.pdf]
